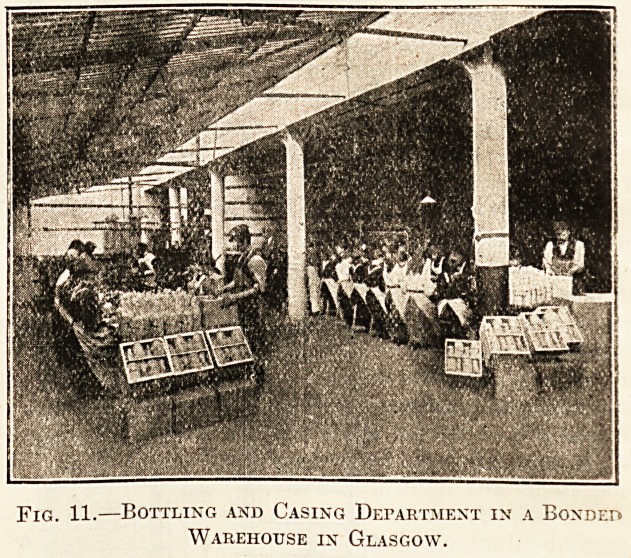# The Hospital Special Analytical Commission on Whisky

**Published:** 1906-04-07

**Authors:** 


### THE HOSPITAL SPECIAL ANALYTICAL COMMISSION ON WHISKY.

Its History, Manufacture, and Properties—Pot-Stills, Patent-Stills, and Blending Illustrated and Described—Whisky in Common Use, with an Analytical Report on Samples of Scotch, Irish, and American Whiskies Purchased Retail in the United Kingdom and the United States of America.

### History.

The preparation of a potable spirit from grain, which is what the manufacture of whisky amounts to, dates back to very ancient times. There is evidence that in the East spirits were distilled from rice and other grain before the Christian era. A spirituous cordial termed "usquebaugh" (Celtic for water of life), from which the word "whisky" is derived, was certainly made in Ireland in the twelfth century. In the preparation of this beverage malt or molasses spirit, sugar, saffron, nutmegs, and other spices were used. Oddly enough, whisky, as we now know it, was apparently originally designated by the term "grain spirit." In the reign of Henry VIII. Irish settlers introduced distillation into Wales in the neighbourhood of Pembroke. The origin of distillation in Scotland appears to be obscure; Scotch spirit was, however, fairly well known in England in the seventeenth century, but we have no exact knowledge concerning the materials employed in its manufacture at this epoch. There is much evidence, however, that grain other than malted barley was used in Scotland in the eighteenth century, and it appears certain that at this time sugar and potatoes were employed for the production of whisky. The above is about all we know concerning the history of the materials from which whisky was made. When we come to inquire into the evolution of the modern distillation technique we find, as in almost every process of evolution, a gradual change from the simple to the complex, each change being introduced to afford a technological advantage—namely, to save labour and expense of production. The primitive still used was a still of the pot-still type, small and greatly resembling the retort of chemists: from this still gradually more and more complicated forms were evolved, the complications for the most part affecting the head of the still and that part of the still lying between the head and the condenser. Fig. 7 is a diagram of a fairly primitive pot-still. Fig. 8

Fig. 1.—Map of Scotland Showing Areas of Production of Different Types of Whisky.

is a photograph of pot-stills at work in a large distillery. In reading the literature of this subject it is very interesting to find what opposition there was to the introduction of large pot-stills, and indeed to almost every change in the type of still-head. In 1830, however, a greater change came, for about this time Coffey introduced the patent-still (see fig. 9). This still is based upon the principles of fractional distillation, and allows of great economy of working on account of the process being continuous, and, on account of less fuel being required. Although much more expensive in the first instance, it has gradually replaced the pot-still, and to-day more than half of the whisky consumed owes its production to this apparatus.

FIG. 3.—GLENTAUCHERS-GLENLIVET, A TYPICAL HIGHLAND DISTILLERY.
By kind permission of Messrs. J. Buchanan and Co., Limited.

### LOCALITY OF MANUFACTURE.

Practically all the whisky consumed in this country is produced in Ireland or Scotland. Irish whisky is known simply as Irish whisky; and although the different makers have slightly different techniques, it is not, as in the case of Scotch whisky, produced in certain districts only, and named after the respective district. Scotch whisky is of five distinct types. Two of these types are produced in the same district. Each of the other types has a district to itself. We have appended a map of Scotland (fig. 1), in which the areas of production of the different whiskies are shaded differently. The types of Scotch whisky are Highland malts, Lowland malts, Islays, Campbeltowns, and grains. Lowland malts and grains are made practically in the same district—that is, in a belt of country between the Frith of Forth and Frith of Clyde, some 20 or 30 miles wide. These various types differ very much from each other, and are quite distinctive, if not always to the chemical expert, at least to the professional taster; individual makes of the same type differ also *inter se*. Substantially all the first four types are made nowadays from a mash consisting entirely of malted barley, but there are some few exceptions. The grain whiskies are made from a mash of malted barley and other malted and unmalted cereal grains, and are distilled in a patent-still. The largest group of Scotch whiskies is the Highland malts, and this group contains several subdivisions, according to the district of production. One of the largest of these subdivisions is Speyside (Glenlivet). We give above a view (fig. 2) of the

FIG. 2.—VIEW OF BRIDGE OVER RIVER SPEY, THE CENTRE OF THE GLENLIVET HIGHLAND MALT WHISKIES.

FIG. 4.—LOCHRUAN, A CAMPBELTOWN DISTILLERY.
By kind permission of Messrs. W. and P. Lowrie and Co., Limited.

bridge made by Stephenson which spans the Spey. We also give a photograph of a typical Highland malt distillery (Glenlauchers-Glenlivet) belonging to Messrs. J. Buchanan and Co., and a Campbeltown distillery (Lochruan) belonging to Messrs. Lowrie.

### Manufacture of Scotch Whisky.

Highland malts, Lowland malts, Islays, and Campbeltowns are all made practically from the same material and in the same way. The only difference is in the type of pot-still used and the way in which it is heated—either by a steam jacket or by fire direct. The by-products in the finished spirit vary according to the type of pot-still used, and hence this is by no means immaterial (*vide infra*). The manufacture of whisky consists of four processes—namely, malting, mashing, fermenting, and distilling. The first operation—namely, malting—is of considerable interest, as the nature of the malt undoubtedly influences to some extent the character of the whisky. The malting in the preliminary "floor" stage is performed in much the same way as in the brewery, and therefore calls for no particular comment in a sketch like the present. It is in the drying off or kilning stage that Scotch distillery practice differs widely from ordinary malting, inasmuch as the element of peat is introduced. According to the type of whisky required more or less peat is employed in kilning the malt. The whiskies in the preparation of which most peat is employed are the Islays. In making the Highland malts less peat is employed, some coke or anthracite being added; for the Lowland malts the amount of peat used is still less—in some cases actually none. In the manufacture of grain whiskies peat is not used at all.

Fig. 5.—Mash Tun in Large Distillery.

The next operation is that of mashing. This consists in converting the starch of the grain into sugar by allowing it to be acted upon by the ferment of the malt, known as diatase. The exact method of doing this, with regard to grinding, temperature, time, filtering, etc., differs at different distilleries. It, however, essentially resolves itself into mixing the ground malt with warm water, in a very large vessel holding many thousands of gallons, and known as the mash-tun. The mash-tun at a large distillery is shown in fig. 5. The result of mashing is to obtain a saccharine liquid which is known as the "sweet wort." The subsequent operation of fermentation is the conversion of the sugar of this saccharine liquid into alcohol. To this end the liquid issuing from the mash-tun is pumped up into a series of large wooden vessels called the fermentation backs (see fig. 6). When each of these vessels is partially filled with it the wort yeast is added, or, as it is technically

Fig. 6.—Fermenting Backs in Whisky Distillery.*

termed, the liquid is *pitched* with yeast. Fermentation is allowed to proceed for about three days, during which time the wort first rises and subsequently falls in the backs. The alcoholic liquid obtained by the fermentation of the wort is known as the wash, and the termination of fermentation is indicated by the specific gravity. The next operation is that of distillation, which has for its object the separation of the bulk of the volatile matters—namely, alcohol—from the non-volatile substances and from a part of the water, and the further rectification of the ensuing alcoholic liquid. Distillation in the case of the Scotch malt whiskies is accomplished in two stages, corresponding roughly to the above-described two phases. The fermented wort is first of all subjected to distillation in a still known as the wash-still.

### Pot-still Whiskies.

In the case of pot-still whiskies the stills used for both these operations are, with the exception of size, the same, and we think here would be a convenient place to describe the pot-still. This form of still varies considerably in size and design. The illustration fig. 7., for which we are indebted to the Distillers' Co., Limited, shows a pot-still of simple construction. It consists of a stout copper vessel, A, set in a brickwork furnace (E being the grate and F the flues), and is surmounted by the copper head B, which is joined up at the bend of the neck to the "lyne-arm," C. The latter is connected directly with the condenser, D. The still shown in this illustration is heated by direct fire, but nowa-

* For this and the preceding illustration we are indebted to Messrs. James Buchanan and Co., Ltd., from whose distilleries they are taken.

days many pot-stills are heated by means of steam jackets or coils. Some pot-stills in Scotland are at the present time fitted with simple rectifying boxes

FIG. 7.—SIMPLE POT-STILL.

or apparatus, and in Ireland the evolution of the pot-still in this direction is very pronounced.

Fig. 8 shows pot-stills at work; the still on the extreme right, which, it will be noticed, is larger than the others, is the wash-still. The two remaining stills are called the low-wines stills, and in them the distillate from the wash-still (termed the low wines) is submitted to a second distillation. This second distillation is essentially a rectification, the distillate being collected usually in three fractions. The first fraction is termed the foreshots, and consists of substances of relatively low boiling-point associated with a good deal of alcohol. The next fraction is the clean spirit, or whisky; the last fraction, relatively poor in alcohol, but containing the substances of high boiling-point, such as oils, ethers, and acids, is termed feints. The first and last fractions are collected in the feints receiver, and are worked up with succeeding charges of low wines. The residue of distillation in the first or wash still is termed burnt ale, that obtained from the whisky-still spent lees; both these liquids are run to waste.

The above process is substantially what takes place in the manufacture of all-malt Scotch whiskies. In Ireland the process differs somewhat; in the first place the mash only very rarely consists of all malt, containing from 25 to 75 per cent. of unmalted grains—namely, barley, rye, oats, and wheat, and exceptionally maize. It would be perhaps convenient here to discuss the use of raw, *i.e.* unmalted, grain as a constituent of the distilling mash. The essential difference between barley malt and barley is that the former contains a ferment called diastase, which is capable of converting the starch of the barley into sugar. We would accentuate the fact that this substance is a ferment, and hence no quantitative relation exists between it and the starch in the grain in which it is produced. In other words, the diastase produced by any given weight of barley

FIG. 8.—POT-STILLS AT WORK.
By permission of Messrs. James Buchanan and Co., Limited.

when it is made into malt is capable of converting very much more starch into sugar than is contained in the malt itself. This fact has been taken advantage of by the Irish pot distiller, who, in the composition of his mash, makes use of the excess of diastase in the malt by presenting it with a certain proportion of unmalted grain to work upon. The second way in which Irish pot-still whisky differs from the Scotch article is in the process of distillation; and this is so both with regard to the shape and size of the still and to the method of working it. In Ireland the fermented wort is distilled at least three times. The Irish stills are also very much larger than the Scotch, and, speaking generally, have much more complicated heads; in other words, the whole process approximates more nearly to the manufacture of grain or patent-still whisky. The whisky is collected at a higher alcoholic strength than is the case with Scotch pot-still whisky. The principle of fractional collection is also rather more complicated.

### Grain Whiskies.

The remaining class of whiskies—namely, the grain whiskies—are made also from a mixed mash, the principle underlying the use of a proportion of unmalted grain in the mash being the same as that described under Irish whisky. The amount of malted barley, however, used is always well in excess of that required to convert the total starch of the mash into sugar. It has been found that an excess of malted barley in the mash is essential for the production of the flavour characteristic of this type of whisky. The mash nearly always contains, in addition to other cereals, some Indian corn, but occasionally all-malt mashes are used for the manufacture of grain or patent-still whiskies. The most striking difference in technique, however, between these whiskies and the pot-still whiskies lies in the apparatus used for distillation. The great technological advantage of the patent over the pot still is that the former brings about in a single operation, and by a continuous process, that which requires several intermittent operations with a pot-still—namely, concentration and rectification of the alcoholic liquid.

### The Patent-still.

The patent-still, of which we give a diagram (fig. 9), was invented by Coffey some seventy years ago, and it is a great tribute to the ingenuity of this inventor that seventy years of technological progress has left this form of whisky-still practically unchanged. Coffey's patent still, as to its main essentials, consists of two communicating vertical columns, each of which is subdivided horizontally into a number of chambers. The column A (see illustration) is termed the rectifier, the column B the analyser. These two columns roughly correspond respectively to the low wines and wash-stills of the pot-still process. Each column, as stated, is subdivided into a series of chambers (*a*, *a*, *a* and *b*, *b*, *b*), the partitions between them consisting of stout copper trays pierced with a large number of small holes. At the commencement of operations steam at a pressure of about 5 lbs. to the square inch is passed in at the base of the analyser (B). Both columns are then filled with steam. When this has been accomplished the wash is pumped up to the top of the rectifier, entering the latter by way of the pipe C. This pipe traverses the whole of the rectifier from top to bottom, passing through each compartment twice—namely, backwards and forwards by means of a double bend. We would here emphasise the fact that the "wash" through its whole course in the rectifier remains in the pipe C. The wash enters the rectifier cold, but by the time it reaches its base it is almost at boiling-point, being heated by the ascending steam and alcoholic vapours coming from the analyser. The wash (still in the pipe C) is then pumped up to the top of the analyser, and there, emerging from the pipe, is discharged on to the tray of the top chamber. The upward pressure of the steam prevents the wash from passing through the small holes which pierce the trays that separate the chambers from one another; each plate is, however, fitted with a drop-pipe standing up about an inch above the level of the tray and passing through the latter into a cup on the plate below. It is through these drop-pipes that the wash, when it has reached a certain height on each plate, can flow on to the next plate, and thus pass from plate to plate from the top to the base of the analyser. The steam entering at the base of the analyser travels upwards, through the small perforations in the trays, and so through the descending wash. By this method the steam gradually deprives the wash of the whole of its alcohol, and the mixed vapours of steam, alcohol, and the other volatile products contained in the wash pass over from the top of the analyser by means of the low-wines pipe *D* to the base of the rectifier. The mixed steam and alcohol vapours then pass up the rectifier, and, in so doing, are gradually cooled. The greater part of the volatile products of a higher boiling-point than the ethylic alcohol (the "fusel oil") is thus gradually condensed, and falls back to the base of the rectifier, whence it is removed by means of a syphon arrangement to a separate vessel (*N*) termed the hot-feints receiver. At a certain part in the upper portion of the rectifying column the temperature approximates to the condensation-point of ethylic alcohol; the tray at this point is termed the spirit plate, from which the condensed alcohol is removed by the pipes *E* or *F*. The spirit plate consists of a tray which differs from those below in that it contains no small perforations, but is pierced by means of one fairly wide pipe only, standing well up above the level of the plate. The part of the rectifier above the spirit plate is cooled in such a manner by the cold wash and by cold-water coils that the alcohol which escapes condensation on the spirit plate is condensed above it and then finds its way back again on to the plate. The alcohol thus condensed is run off, according to its purity, either by *E* or *F*, to the spirits or feints receiver. Those volatile substances in the wash which have a lower boiling-point than the ethylic alcohol, such as the aldehydes, pass out at the top of the rectifier by the pipe *H*, and are thence conducted to a separate condenser in the trough *G*. Here the aldehydes, etc., condense, and can be separately stored, or, as is frequently the case, are returned to the still for further rectification. In the hot-feints receiver (*N*) the fusel oil separates from the main solution; this latter, in order that no alcohol may be lost, is pumped back to the top of the analyser. The fusel oil is finally collected in the fusel box (*P*). The working of the still is controlled by an operator stationed on the floor-level at a point opposite to the sampling stage

Fig. 9.—Diagram of a Coffey Patent Still, Cambus Distillery.
By permission of the Distillers' Co., Limited.

(*K*). Here, by means of sampling glasses, he can obtain samples of the various products entering or leaving either of the two columns, and is thus able to observe whether the wash leaving the base of the analyser is entirely deprived of its alcohol, what the strength of the spirit leaving the spirit plate is, and the strength of the vapours passing over through the low-wines pipe, etc. According to the indications thus received, he will cause the wash to travel a little faster or slower, or turn steam on or off, or run the whisky into the feints instead of into the spirits receiver, and so on. It will be observed that although the patent still is very different in appearance to the primitive pot-still, yet the principle of pot-still and patent-still manufacture is essentially the same. Low wines, feints, and foreshots, etc., are obtained in both processes, the main difference being that in the patent-still the process is continuous and is finished in a single operation, whereas in the pot-still several operations are required. The finished product of the patent-still differs to some extent chemically from that of the pot-still; but before we consider this in detail we think it advisable to describe three processes which occur to the products of both stills before they are consumed by the public; these are ageing, blending, and bottling.

Whisky, of whatever kind, when it leaves the still is undrinkable. To get rid of or to modify the substances which render it nauseous it is stored for varying times. This storing is accomplished in oak casks of various kinds. The casks are of three main types—namely, fresh sherry cask, refill cask, and plain wood cask. This process is termed maturation, and the period required for its satisfactory accomplishment depends entirely on the type of spirit. The heavy, rich Islay whiskies require considerably more time to mature than the Speyside (Glenlivet) Highland malts; and the latter, in their turn, do not ripen as quickly as the Lowland malts. The grain (patent still) whiskies finally are potable and agreeable sooner than any of the other varieties mentioned. It is somewhat difficult to say, therefore, how old a whisky should be before it may be correctly, in the commercial sense, described as mature; but there appears to be a consensus of opinion among expert tasters that a minimum age of about two to three years is desirable even for the lightest types. That there is a good deal of immature whisky on the market cannot be gainsaid, and our examination (see below) of a number of draught whiskies from low-class public-houses confirms this fact; at the same time our inquiry has led us to believe that the great bulk of the whisky sold by respectable firms is of reasonable quality and age. We shall treat the subject of ageing from the chemical and physiological side below.

### Blended Whisky.

The vast majority of whisky drunk to-day is so-called *blended* whisky. It is only very rarely that a whisky which is solely the product of one distillery, whatever be its nature, is suited to the public taste, which must be the eventual criterion of the commercial value of a whisky. The public will have

Fig. 10.—Blending Department of a Bonded Warehouse in Glasgow.

the whisky it likes, and the popular whisky of the present day is undoubtedly a blended whisky, *i.e.* a spirit obtained by judiciously putting together a number of whiskies of varying ages and types, so as to produce an article at a given price which the expert blender knows will be acceptable to the public. Blending has now become a fine art, and there is no doubt that it is this art which has made Scotch whisky the most popular spirit of the day. The blends which are most sought after by the public are those composed of a number of mature whiskies representing the various types of pot-still whiskies, together with a fair proportion of grain,

Fig. 11.—Bottling and Casing Department in a Bonded Warehouse in Glasgow.

*i.e.* patent-still, whisky. The value of grain whisky as a component of blends is now well established, and cannot be regarded as dependent upon its relatively lower price. It is claimed for it, and from our research we think with justice, that by its use the flavours of the malt constituents of the blend are made to harmonise better, that it is lighter and less liable to disagree, and that it enables the blender to give better value for a given price with regard to the age and quality of the malt whisky used. The illustration (fig. 10) * shows the interior of a large blending warehouse in Glasgow. Blending is generally conducted in bond; the operation is performed in large vats holding as much as 20,000 gallons. This blending is by no means a simple affair. All the whiskies of the same class are first carefully mixed together in the required proportions, and then each mixture is added separately to the blending-vat. When all the different whiskies have been put into the vat, its contents is intimately mixed by a powerful spray of compressed air.

The last process which we have to describe is that of bottling, of which we append an illustration, as it is conducted in a bonded warehouse. The labelling of whisky is at present a much discussed question. We think that within limits the consumer is entitled to know what he is buying, and that the label should be an honest one. For instance, a whisky should not be called Highland malt if it is a blend of Highland malt and Lowland malt; nor should it be called pure malt if it be a blend of malt and grain. On the other hand, it is not fair to expect the blending trade to disclose the composition of popular blends which are obviously valuable

* For this and the succeeding illustrations we are indebted to Messrs. J. Buchanan and Co., Ltd.

trade secrets and have cost much time and money to acquire.

Chemistry and Therapeutics.

Having considered the technical processes employed in the manufacture of whisky from malting to bottling, we now come to discuss what is known of the chemistry and therapeutics of the finished article. The final products of the pot and patent still differ chemically to some extent both from each other, and also *inter se*. It would perhaps be better to pause here in order to define another trade term which is often used loosely and often inaccurately—namely, "silent spirit." What silent spirit really means is a spirit, *i.e.* a mixture of ethylic alcohol and water of such purity *i.e.* rectified to such a degree as to contain either no impurities at all, or an insufficient number to indicate the origin of the spirit; hence the term "silent," which really means silent as to its origin. Unfortunately this term "silent spirit" has been applied generically to all spirits produced in a patent or Coffey still, as distinguished from those produced in a pot-still. It must be at once emphasised in this connection that such spirits are certainly not necessarily, and practically never are, silent.

Blended whisky occupies chemically an intermediate place between pure pot and pure patent still whisky. Although whisky differs in alcoholic strength according to the method of distillation, and hence in different districts and distilleries, yet nevertheless as it reaches the consumer this strength is practically constant. Hence the main difference between different kinds of whisky from the chemical standpoint arises from the fact that different whiskies contain different amounts of so-called impurities. Before entering into this subject it should be at once pointed out that although there is no doubt that the amount of these so-called impurities modifies the taste of a given whisky, yet nevertheless in the present state of our chemical knowledge we cannot refer the characteristic taste and flavour of whisky to any one, or indeed to any group, of these impurities. The impurities—or, perhaps better, by-products—in whisky are present in very small quantity, even in those whiskies which are relatively very rich in them; roughly 200,000 parts of whisky at proof strength contain from 100-300 parts of impurities. The impurities, although differing very largely among themselves both chemically and therapeutically, are sometimes classified by the analyst together, and the sum of them all is termed the coefficient of impurities. They consist of substances originally present for the most part in the mash or wort, the physical properties of which (volatility, etc.) render it difficult to separate them completely from alcohol. Chemically they consist of higher alcohols or alcohols having a higher molecular weight than ethyl alcohol—namely, propyl, butyl, and amyl alcohols—the so-called fusel oil, compound ethers, or substances chemically allied to ethyl acetate or the acetic ether of the British Pharmacopœia, and aldehydes, or substances built up on the lines of the official hypnotic paraldehyde. Amongst the aldehydes is found a substance called furfural, which has attracted a good deal of attention chiefly on account of the fact that it gives an easily obtained and very distinct colour reaction, and that it is, as far as we know, the most toxic constituent of whisky. Besides the substances mentioned above, other bodies, concerning the chemical nature of which we are ignorant, are undoubtedly present, and it is probably to them that whisky owes its characteristic taste and flavour.

Before entering into the question of the therapeutic effect of whisky we would emphasise the fact that it is a spirit, *i.e.* a product of distillation, and therefore contains no living organisms; and, further, its alcoholic strength is such as to keep it absolutely sterile. Thus, in common with all spirits, any change occurring in it after it leaves the still is due to chemical or physical processes, and this quality distinguishes it from wine or beer. Whisky differs from brandy in taste and flavour, also in that it contains relatively more higher alcohols and relatively less compound ethers; it further contains traces of so-called empyreumatic or tarry substances derived from the malting processes.

The main constituent of whisky from the standpoint of the therapeutist is unquestionably alcohol, and this constituent is not affected by the materials from which whisky is made. The British Pharmacopœia does not contain whisky among the alcoholic preparations, but the American Pharmacopœia does, and at the same time dictates the materials from which whisky can be made. In these directions, as a matter of fact, very slight restrictions are placed either upon the composition of the mash or upon the diastatic agent to be used to convert its starch into sugar. It is expressly stated that maize or Indian corn may be used for the mash, and it is not stated that even a small proportion of malt must be employed. The chief advantage of whisky over alcohol as a therapeutic agent is its flavour, whisky giving the physician alcohol in a palatable form. Concerning the therapeutic action of the so-called impurities in whisky—*i.e.* the higher alcohols, the ethers, and aldehydes—we have very little exact information. The dictum found in several text-books concerning the therapeutic value of these substances and the *rôle* they play in modifying the therapeutic action of the alcohol present does not, upon careful investigation, seem to rest upon any very sound experimental or clinical evidence. There is certainly reason to think that the impurities, although placed together by the analyst (the so-called coefficient of impurities), differ therapeutically *inter se*. What evidence we have points to the conclusion that, apart from their taste, the higher alcohols and the aldehydes have an action the same in kind as ethylic alcohol, only weight for weight are more active. The action of the compound ethers probably differs to some extent from that of alcohol, and they may be regarded in the present state of our knowledge as stimulants. The present paper, or indeed one many times its size, would be far too small to discuss adequately the therapeutic action of alcohol or whisky. At the beginning we would draw attention to the question as to whether this subject belongs to therapeutics or to dietetics; in other words, whether alcohol is more properly described as a drug or as a food. In considering alcohol from this standpoint we must always remember that it not only modifies the behaviour of certain organs as a drug, but also actually supplies energy to the body as a food. Somewhere about 90 per cent. of any moderate quantity of alcohol taken is burnt up by the body—that is, converted in $CO_2$ and $H_2O$—and in this conversion energy is evolved. The remaining 10 per cent. is excreted unchanged, and this moiety is not a source of energy. We frequently hear alcohol spoken of as a stimulant, and in so far as this term simply means an agent which increases the activity of any given organ it is accurate. Stimulants, however, from the therapeutical standpoint, are agencies which increase the activity of organs by unlocking some energy already present in the organ, and in the case of alcohol, if this action exists at all, it is certainly subordinate to the effect of alcohol in increasing the amount of labile energy available. The specific system upon which alcohol acts as a drug is the nervous system, and with regard to its effect on this system authorities are not in accord, some regarding its action as at first stimulant, *i.e.* increasing the action of the nervous centres, and finally depressant—that is, paralysing them; other therapeutists deny that any even ephemeral stimulating action is excited by it, asserting that the action is depressant throughout. Both these views seem supported by considerable evidence, but there can be no doubt that the main action of alcohol as a drug upon the nervous system is depressant. The glib conversation which often gives place to the awkward silence at a dinner-party after the first few glasses of wine have been drunk seems superficially to militate against the *ab initio* depressant action of alcohol; really it does not do so, for the silence is due to activity of the highest of all nervous centres—namely, those producing control, or what physiologists term inhibitory. It is to the paralysing or depressant action of alcohol upon these centres that the free conversation in question is due, not to an actual stimulating action upon the lower or speech sensori-motor centres. The depressant action of alcohol upon the nervous system is from top to bottom; it first depresses the highest inhibitory centres, then the sensori-motor cortical centres, then the spinal cord, and finally the respiratory and cardiac centres, or those situated in the medulla oblongata.

### Whiskies in Common Use.

Having considered the manufactures and properties of whisky in general, we now come to that part of our inquiry which was directed to ascertain by direct investigation the nature of the actual whiskies sold to the public. In this connection we have examined, firstly, whiskies on the home market; secondly, home whiskies exported and sold in America; and, thirdly, in order to compare the quality of the whisky exported to this most important market with the domestic article sold in that country, a number of American (rye) whiskies.

Before describing the spirits taken for the purposes of investigation, we propose to enumerate the points to which we more particularly directed our attention:—

1. Whether the samples consisted of genuine Scotch or Irish whiskies, respectively, and if so, what was the general nature of the article.

2. Whether the samples contained anything liable to be injurious to health.

3. Whether the sample was mature—*i.e.* properly aged.

4. Whether the sample was of a fair price considering the quality.

5. Whether the samples were in any way misdescribed.

*The home samples* were collected by ordinary purchase by the special Hospital commissioner. Seven of these were Scotch and seven Irish. Of each lot four were bottled whiskies of well-known brands, and three were draught whiskies collected in the neighbourhood of the docks in the East End of London. In the tables showing the general analytical results, *A*, *B*, and *C* are the bulk Scotch whiskies, and *H*, *J*, and *K* the bulk Irish whiskies; the others—namely *D*, *E*, *F*, *G*, and *L*, *M*, *N*, *O*—are the Scotch and Irish bottle whiskies respectively. In addition to the analytical results we give the price in shillings per proof gallon of the article.

Table I.

*Scotch Whiskies.*

| Sample | Alcohol per cent. by Vol. | Extract per cent. | Ash per cent. | Total Acid | Non-Volatile Acid | Volatile Acid | Ethers | Higher Alcohols | Aldehydes | Furfural | Approx. Price per Proof Gallon in Shillings |
|---|---|---|---|---|---|---|---|---|---|---|---|
| *A*.... ... ... | 45.01 | .3336 | ·0128 | 13.8 | 0.8 | 13.0 | 41.6 | 182 | 12.0 | 1.0 | 27.9 |
| *B*.... ... ... | 43.07 | .108 | .016 | 17.2 | 0.83 | 16.37 | 58.6 | 95 | 12.7 | 2.08 | 29.2 |
| *C*.... ... ... | 42.23 | .164 | .0144 | 23.8 | 3.5 | 20.3 | 26.6 | 77 | 17.1 | 0.9 | 31.0 |
| *D*.... ... ... | 47.45 | .096 | .0055 | 42.4 | 10.5 | 31.9 | 48.5 | 92 | 18.9 | 2.6 | 28.8 |
| *E*.... ... ... | 47.69 | ·1248 | .0144 | 31.7 | 7.9 | 23.8 | 57.4 | 193 | 24.7 | 3.1 | 27.2 |
| *F*.... ... ... | 47.56 | .388 | .018 | 79.2 | 32.8 | 46.4 | 64.3 | 157 | 8.6 | 3.17 | 25.2 |
| *G*.... ... ... | 46.98 | .1684 | .0100 | 59.1 | 15.9 | 43.2 | 58.2 | 146 | 18.0 | 3.4 | 29.1 |
| | | | | | *Irish Whiskies.* | | | | | | |
| *H*.... ... ... | 44.27 | .1776 | .008 | 35.2 | 8.8 | 26.4 | 31.08 | 90 | 12.1 | trace | 24. |
| *I*.... ... ... | 41.30 | .0976 | .0144 | 5.8 | 3.6 | 2.2 | 22.1 | 93 | 14.5 | 0.4 | 29.4 |
| *K*.... ... ... | 38.98 | .0976 | .014 | 6.1 | 3.1 | 3.0 | 34.3 | 70 | 8.2 | nil | 17.5 |
| *L*.... ... ... | 45.99 | .088 | 0.004 | 5.2 | 2.6 | 2.6 | 22.2 | 76 | 15.2 | trace | 23.5 |
| *M*.... ... ... | 46.10 | .092 | .009 | 20.8 | 5.2 | 15.6 | 40.1 | 170 | 15.1 | 1.1 | 32.2 |
| *N*.... ... ... | 47.95 | .0736 | .0048 | 26.0 | 6.2 | 19.8 | 23.4 | 351 | 10.8 | 3.4 | 28.5 |
| *O*.... ... ... | 51.57 | .124 | .0012 | 29.3 | 9.3 | 20.0 | 40.6 | 217 | 12.2 | 4.0 | 22.1 |

*Sample A.*—The analytical figures indicate that this is a medium to low class spirit of little age. The taste of the sample was decidedly unpleasant and new, and indicated the possibility that the sample contained a certain amount of spirit which cannot be fairly described as Scotch whisky—*i.e.* neither grain nor malt.

*Sample B.*—Was somewhat superior to *A*, and in this case there is no reason to suspect that the sample was misdescribed. The sample, however, was of poor quality and scarcely mature.

*Sample C.*—Was almost characterless in flavour, and the analytical numbers taken in conjunction with the taste of the sample indicate a spirit consisting of a little pot-still spirit blended with a considerable proportion of patent-still spirit—the latter, perhaps, not Scotch grain whisky.

To sum up the bulk (" draught ") Scotch whiskies, they are—in view of the price charged—of poor quality. At the same time, apart from the fact that their maturity is scarcely what is desirable, there is nothing to show that these samples contain anything that might be described as being injurious to health. One of the samples, at least, appears to us to be probably misdescribed.

### The Scotch Bottle Whiskies.

Both from the point of view of the analytical figures and of taste, it is quite obvious that these whiskies are incomparably superior to the bulk whiskies referred to above. As a class they may all be described as being fair value for money, and it is pleasant to be able to say that not a single one of these whiskies presents any objectionable feature of quality. Sample *D*, perhaps, is not in some respects of so high a quality as the others, but it is obviously a well-matured Scotch whisky, although we are inclined to think that the word " Highland," which is used on the label, does not accurately represent the whole composition of the whisky. It would scarcely be fair to compare the high-class bottle whiskies with one another, for here a preference is obviously entirely a mater of individual taste. In this connection we would emphasise the importance from a therapeutical standpoint of the taste of the whisky. The only action that whisky can have as compared with an equal quantity of equally dilute ethylic alcohol upon digestion must depend upon the reflex influence it can exert upon the digestive and perhaps other functions through the palate. The enormous difference in the way different individuals are affected by the same smell and taste is proverbial, as are also the delightful and horrible effects which may be occasioned by these senses. In the present state of our chemical knowledge we cannot, in whisky point definitely to the chemical bases of taste and flavour. Substances escaping at any rate quantitative chemical analysis certainly influence, if they do not actually endow whisky with, these properties. The public may be educated with regard to taste, and the function of the expert taster may have an ethical value. The taste and flavour characteristics of the Scotch bottle whiskies as they appeared to us are as follows:—

*Sample D* is somewhat light, and has some elegancy, but with little body. The flavour is distinctive and in some respects resembles Irish whisky. It is quite mature.

*Sample E.*—Full in body and flavour, with a slight grip on the palate which is no doubt a pleasing feature to many consumers.

*Sample F* is a very big or fat whisky, with much flavour and body. This whisky will appeal to those who prefer a very pronounced flavour.

*Sample G* has body, elegance and flavour, combined with a full maturity. This whisky appears to strike the happy mean between the powerful, excessively peaty types on the one hand, and the excessively thin or elegant types on the other hand.

With regard to price, it will be noted that the superior bottle whiskies average less than the inferior public-house " bulk " whiskies.

### The Irish Whiskies.

With regard first to the Irish bulk whiskies it may be said that as a class they are distinctly inferior to the Scotch bulk spirits, although the latter were certainly not—always keeping in view the *price* as the main criterion in this connection—of high quality.

*Sample H* is poor in quality, but possesses some of the characteristics of Irish whisky. We doubt, however, whether it consists entirely of Irish spirit.

*Sample J* is decidedly better than either of the other bulk whiskies, but appears to lack age.

*Sample K* is certainly low in price, but this appears to be its only claim to merit. It is crude and inferior.

*The Irish bottle* whiskies are all of high quality excepting *L*, which is somewhat poor—but at worst may be described as inoffensive.

*Sample M* is of the light and elegant type; Samples *N* and *O* are much bigger or fatter whiskies with full body and flavour.

*Summary.*—On the whole, the quality of the bottle whiskies representing well-known brands is thoroughly satisfactory, and the price demanded a fair one. The most striking feature is the superiority of the bottle to the bulk whiskies considering the fact that the price paid is practically the same. At the same time it must be remembered that the bulk whiskies were purposely collected in a district which was likely to yield the most inferior quality of spirits, and that these, therefore, probably represent the low-water mark of what is sold to the public. In this connection, therefore, it is some satisfaction to note that the worst that can be said of these bulk whiskies from low-class public-houses is that they are poor in quality, occasionally that they are misdescribed, and that they do not possess the full maturity that is desirable. We were not able to confirm the statements as to the sale of actually poisonous concoctions, nor to obtain evidence of anything likely to produce a worse effect than a headache or a disordered stomach. Where quality pure and simple is concerned we fear that the consumer must learn by his own experience, and if he cares to give an excessive price for an inferior article, the old adage " caveat emptor " must if needs apply, but at the same time we think that a compulsory age limit might be desirable. If the consumer wants quality at a fair price our investigation will afford him the opportunity of discrimination, inasmuch as if he insists on having the bottle goods of some respectable firm—and there is no reason why this should not be done in even the poorest districts—he will get what he asks for. It is interesting to note that a similar inquiry to the one above was instituted by the Select Committee on British and Foreign Spirits and the results of it contained in the report of this Committee are practically the same as our own. In our case, however, fuller chemical analyses have been made.

The second part of our experimental investigation is concerned with whiskies obtained from America, consisting of twelve samples kindly procured by Sir Henry Burdett. We received no history of these whiskies, but our investigation has convinced us that eight of these samples are Scotch whiskies, imported to be sold in the United States, and that the remaining four whiskies are of American manufacture. The analytical results are shown in Table II.

In connection with our description of the American samples it may be of interest to briefly refer to the manufacture of whisky as practised in America. Roughly speaking, there are in America two main classes of whisky—namely the "rye" whisky and the "Bourbon" whiskies. The mash from which the former are made consists chiefly of rye; that from which the latter are made, of maize (Indian corn). The diastatic agent in both cases is malted grain. In America, as in this country, whisky is made both by means of the pot and patent still. The apostles of the pot-still product have given it the name of "straight," with the object, no doubt, of depreciating blended and patent-still whisky; as a matter of fact, the name does not seem to have done much for the product, in that only about from 10 to 20 per cent. of all whisky sold in America is straight whisky.

With regard to the American samples (Nos. 7-10 in the above table) that we examined, it is, of course, not possible to compare these directly with the Scotch samples, as the character of the two types is so radically different; nevertheless, we are of opinion that the American whiskies are not of quite so high a class of spirit as the Scotch samples. At the same time, there is nothing to indicate that these whiskies are not derived from a fair class of material, nor that they are of insufficient age or in any way injurious to health.

Table II.

*Whiskies from the United States of America.*

| Sample No. | Alcohol per cent. by Vol. | Extract per cent. | Ash per cent. | Total Acid | Non-Volatile Acid | Volatile Acid | Ethers | Higher Alcohols | Aldehydes | Furfural |
|---|---|---|---|---|---|---|---|---|---|---|
| 1 | 46.90 | 0.10 | 0.007 | 49.3 | 19.7 | 29.6 | 65.2 | 93.9 | 10.8 | 2.25 |
| 2 | 46.90 | 0.10 | 0.008 | 53.6 | 20.9 | 32.7 | 65.2 | 88.0 | 10.8 | 2.25 |
| 3 | 46.99 | 0.15 | 0.008 | 63.8 | 27.0 | 36.8 | 60.8 | 112.0 | 16.3 | 3.19 |
| 4 | 46.70 | 0.13 | 0.008 | 64.0 | 29.5 | 34.5 | 61.2 | 113.0 | 16.6 | 3.20 |
| 5 | 46 16 | 0.14 | 0.008 | 49.3 | 24.8 | 24.5 | 68.4 | 85.0 | 10.1 | 3.00 |
| 6 | 46.03 | 0.14 | 0.009 | 49.5 | 24.9 | 24.6 | 68.6 | 78.7 | 10.4 | 3.00 |
| 7 | 47.75 | 0.22 | 0.008 | 84.4 | 55.5 | 28.9 | 84.5 | 80.3 | 8.4 | 2.90 |
| 8 | 47.69 | 0.21 | 0.007 | 87.5 | 59.1 | 28.4 | 88.5 | 78.0 | 10.7 | 2.80 |
| 9 | 46.60 | 0.40 | 0.010 | 87.5 | 34.7 | 52.8 | 76.0 | 87.0 | 8.8 | 3.00 |
| 10 | 46.70 | 0.40 | 0.013 | 82.2 | 33.4 | 48.8 | 69.7 | 68.7 | 9.9 | 2.50 |
| 11 | 48.10 | 0.18 | 0.008 | 64.8 | 31.2 | 33.6 | 66.6 | 78.0 | 18.0 | 3.30 |
| 12 | 47.97 | 0.18 | 0.009 | 65.0 | 31.2 | 33.8 | 58.7 | 86.0 | 18.7 | 3.30 |

Note.—In both of the above tables (1 and 2) the results (excepting those for the alcohol, extract and ash) are expressed in parts per 100,000 part of absolute alcohol. The higher alcohols have been estimated by the Allen-Marquardt method.

### Comment on the Samples from America.

With regard to, first of all, the samples of imported Scotch whisky, it is gratifying to be able to say that all these samples are obviously—judged by analysis and also by taste—whiskies of good quality and of mature age. In view of the statements current in America, the result of the examination of these samples, which are very fairly representative of the class of Scotch whisky exported to the States, possesses a very special interest. With regard to the merits of the individual whiskies, Nos. 1 and 2 represent a somewhat light type of spirit, though possessing quality, age and elegance. Nos. 3 and 4 are whiskies of a fuller type, of excellent quality all round, possessing body, elegance of flavour, combined with considerable age. Nos. 5 and 6 are, as regards their general character, intermediate between Nos. 1 and 2, and 3 and 4. Nos. 11 and 12 are perhaps the samples which are most in accordance with the general run of the public taste. Neither too full nor yet over-light, they combine with a sufficiency of body delicacy of flavour and full age.

### Summary.

Practically the last published report of an investigation upon whisky was that of the Select Committee on British and Foreign Spirits which appeared in 1891. If we compare roughly our conclusions with theirs, we find that there is no substantial difference between the two, although fifteen years have elapsed since the former publication. Speaking generally, in our opinion, whisky has earned its reputation as a hygienic beverage, and the article supplied to the public is as a rule of good quality. The only exception to this appears to be the increase in the sale as draught whisky of imperfectly aged spirit. We cannot say that we have found in these spirits any ingredient likely to be injurious to health, but injury from such causes is often exceedingly difficult to prove. This question of the injuriousness to health of immature whisky and also the nearly allied one of the relative therapeutical values of different k'nds of whisky is a field of research likely to yield results important both to the hygienist and to the practical physician. Especially is this so as we have now at our command methods of experimental research and clinical observation less available when the last investigation was made and highly likely to yield definite answers to these important questions.

## Figures and Tables

**Fig. 1. f1:**
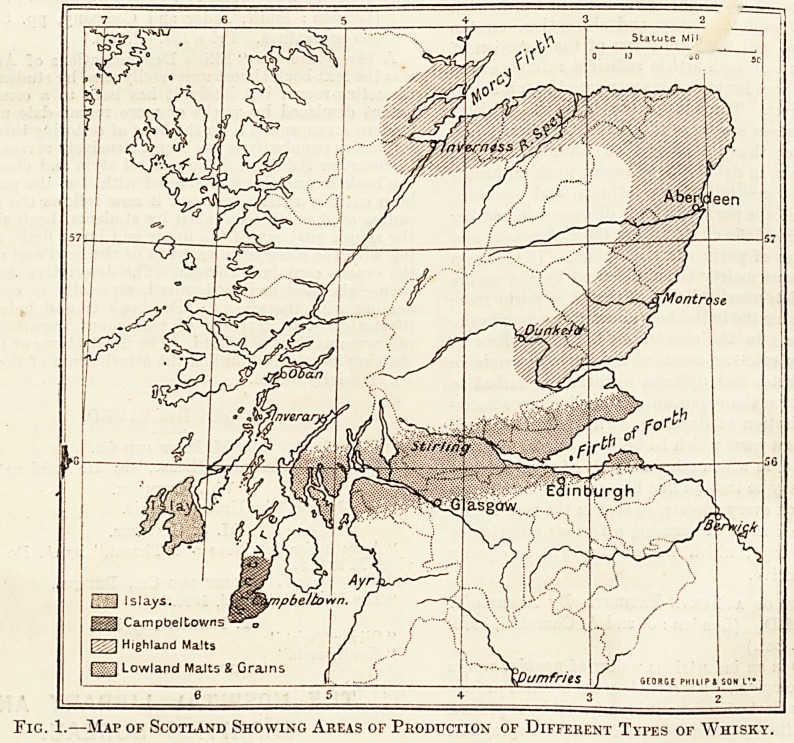


**Fig. 2. f2:**
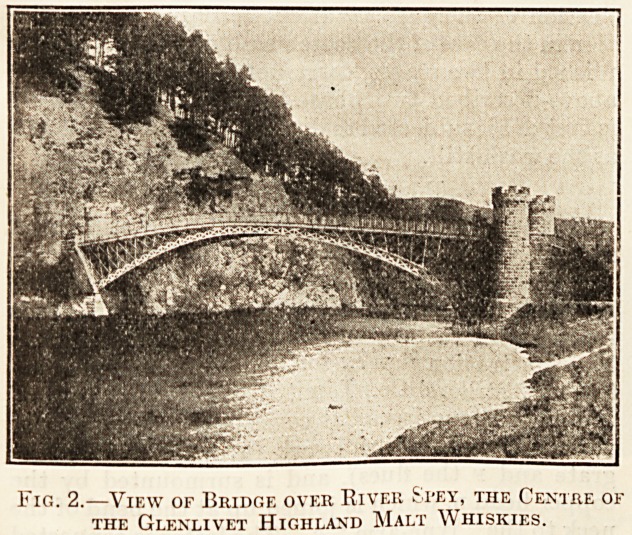


**Fig. 3. f3:**
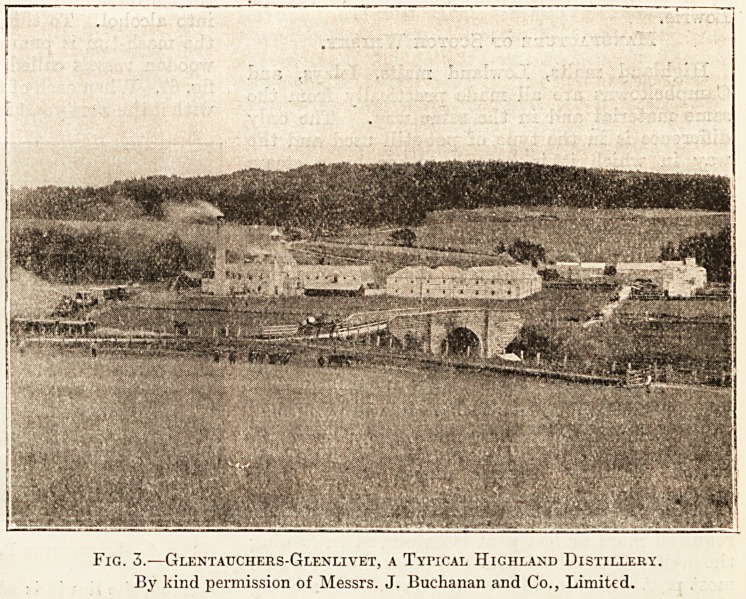


**Fig. 4. f4:**
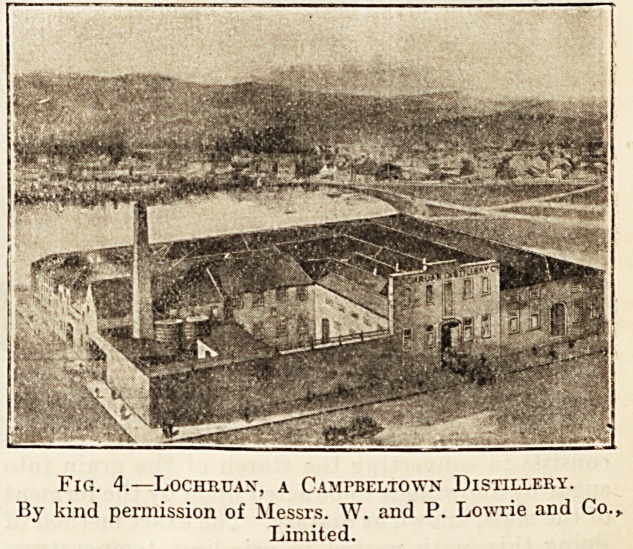


**Fig. 5. f5:**
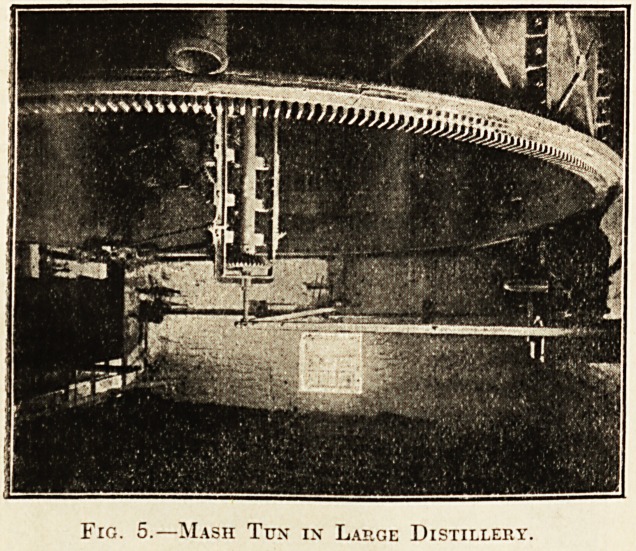


**Fig. 6. f6:**
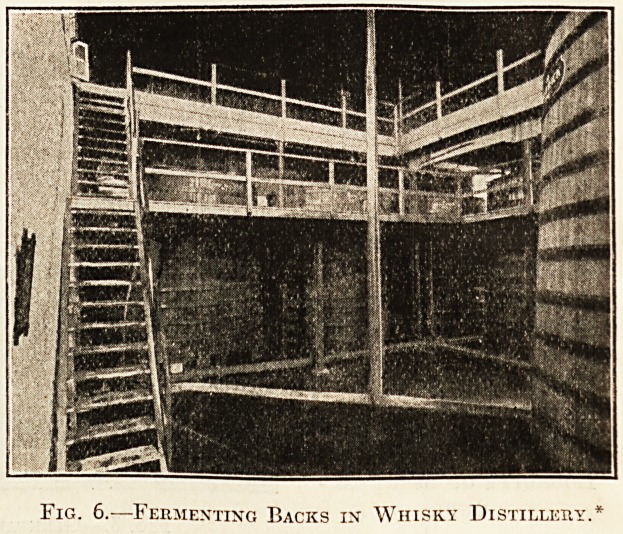


**Fig. 7. f7:**
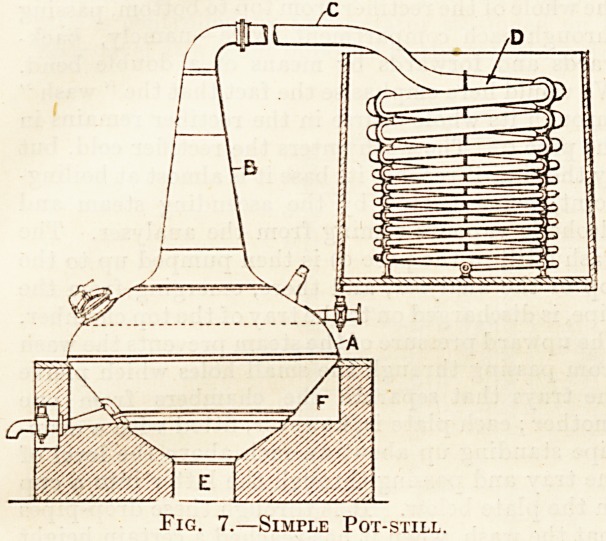


**Fig. 8. f8:**
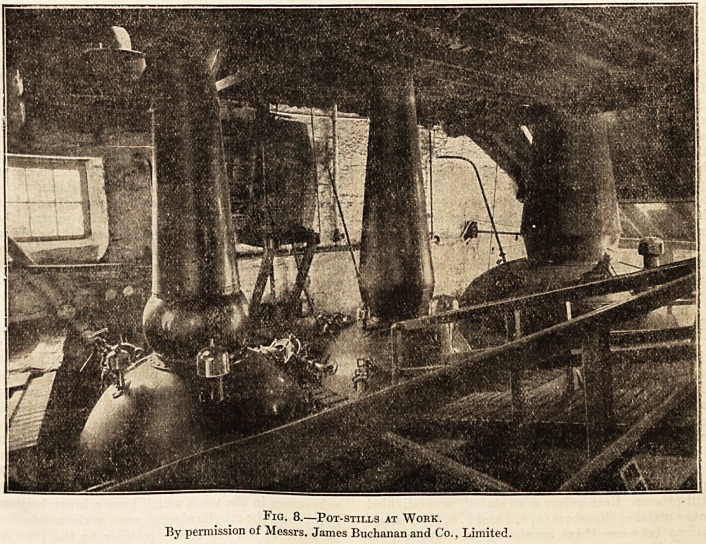


**Fig. 9. f9:**
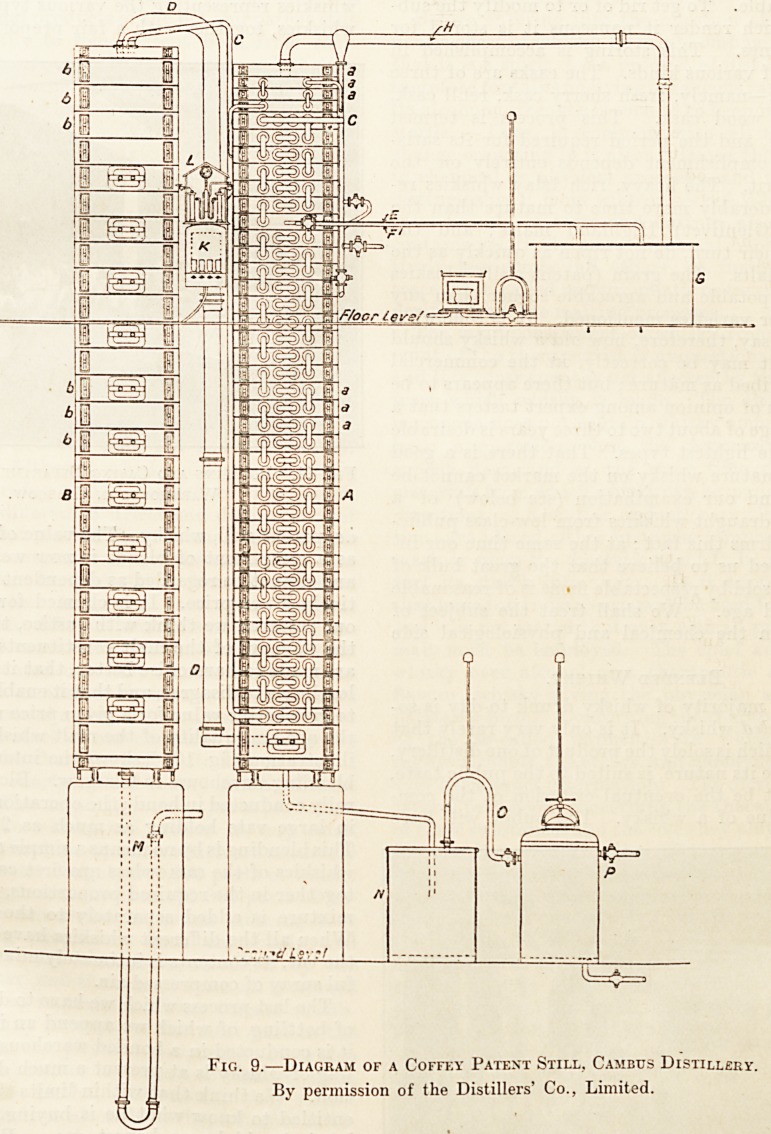


**Fig. 10. f10:**
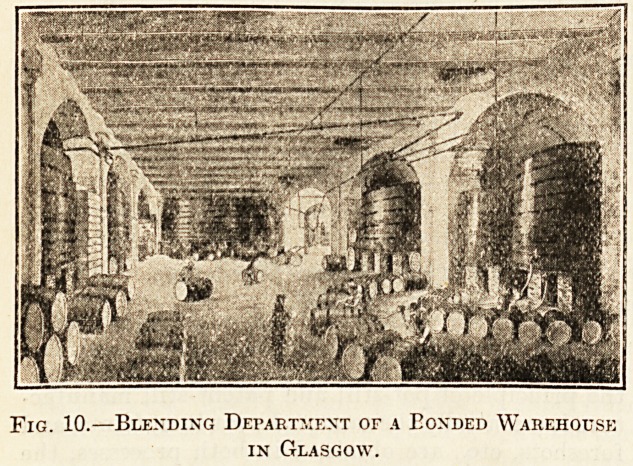


**Fig. 11. f11:**